# Involvement of matrix metalloproteinases (MMPs) and inflammasome pathway in molecular mechanisms of fibrosis

**DOI:** 10.1042/BSR20160107

**Published:** 2016-07-15

**Authors:** Sacha Robert, Thomas Gicquel, Tatiana Victoni, Samuel Valença, Emiliano Barreto, Béatrice Bailly-Maître, Elisabeth Boichot, Vincent Lagente

**Affiliations:** * UMR991 INSERM, Université de Rennes 1, 35043 Rennes, France; † Laboratório de Reparo Tecidual, DHE/IBRAG/UERJ, 20550-170 Rio de Janeiro, Brazil; ‡ Instituto de Ciências Biomédicas, Universidade Federal do Rio de Janeiro, 21941-902 Rio de Janeiro, Brazil; § Laboratory of Cell Biology, Federal University of Alagoas, 57072-900 Maceió-AL, Brazil; ¶ INSERM, U1065, Faculté de Médecine, Université de Nice Sophia Antipolis, 06204 Nice, France

**Keywords:** fibrosis, inflammasome, interleukin-1, liver, lung, matrix metalloproteinase, purinergic receptors, tissue inhibitors of metalloproteinase (TIMP)-1

## Abstract

Fibrosis is a basic connective tissue lesion defined by the increase in the fibrillar extracellular matrix (ECM) components in tissue or organ. Matrix metalloproteinases (MMPs) are a major group of proteases known to regulate the turn-over of ECM and so they are suggested to be important in tissue remodelling observed during fibrogenic process associated with chronic inflammation. Tissue remodelling is the result of an imbalance in the equilibrium of the normal processes of synthesis and degradation of ECM components markedly controlled by the MMPs/TIMP imbalance. We previously showed an association of the differences in collagen deposition in the lungs of bleomycin-treated mice with a reduced molar pro-MMP-9/TIMP-1 ratio. Using the carbon tetrachloride (CCl_4_) preclinical model of liver fibrosis in mice, we observed a significant increase in collagen deposition with increased expression and release of tissue inhibitors of metalloproteinase (TIMP)-1 both at 24 h and 3 weeks later. This suggests an early altered regulation of matrix turnover involved in the development of fibrosis. We also demonstrated an activation of NLRP3-inflammasome pathway associated with the IL-1R/MyD88 signalling in the development of experimental fibrosis both in lung and liver. This was also associated with an increased expression of purinergic receptors mainly P2X_7_. Finally, these observations emphasize those effective therapies for these disorders must be given early in the natural history of the disease, prior to the development of tissue remodelling and fibrosis.

## INTRODUCTION

Fibrosis is a basic connective tissue lesion defined by the increase of the fibrillar extracellular matrix (ECM) components in a tissue or organ. It is a frequent component of a chronic inflammatory process but can also occur in other pathological conditions (vascular, metabolic, tumour pathologies). Sclerosis is related to fibrosis tissue induration. It is therefore a term macroscopic but often used as a synonym for fibrosis. The ECM is a multimolecular complex structure comprising fibres of collagen, elastic fibres, glycoproteins of structure including fibronectin and laminin, and mucopolysaccharides. It is organized into a 3D network and physiologically in balance between the processes of synthesis, deposit in the extracellular environment and the process of degradation of these molecules. The constitution of fibrosis is the result of a disruption of the balance of the ECM: increase of process synthesis and deposition of the components of the ECM on a hand and decrease of their degradation on the other hand. Incorporated fibrosis may remain stable, worsen under the action of tissue attacks, or regress. Regression is a rare development, regarding recent fibrosis and requiring the disappearance of the initial stimulus of the fibrogenesis. If prolonged chronic inflammatory reaction spontaneous evolution is often fibrosis but not all fibrosis are inflammatory.

The aim of the present review is to analyse the involvement of the matrix metalloproteinases (MMPs) and the inflammasome pathway in the physiopathological development of two types of fibrosis: pulmonary fibrosis and liver fibrosis.

Pulmonary fibrosis is a severe and crippling disease with a poor prognosis. Its main histological features include alveolar septal lesions, abnormal reepithelialization, fibroblast proliferation and excessive deposition of ECM macromolecules due to abnormal wound healing, and inflammation characterized by an influx of macrophages, neutrophils and lymphocytes. Idiopathic pulmonary fibrosis (IPF)–usual interstitial pneumonia–is the most frequent form of interstitial pneumonia of unknown aetiology [1,2]. It has been assumed that IPF is the consequence of chronic inflammation [3]. Nevertheless, treatment with glucocorticoids, which are the most potent anti-inflammatory drugs, did not have the expected improving effects on the development of pulmonary fibrosis [4]. Thus, the links between inflammation and fibrosis have been extensively debated and need to be clarified [1,5].

Hepatic fibrosis develops following chronic inflammatory process under the influence of repeated stimulation. Its progress may cause the occurrence of cirrhosis or cancer of the liver, one of the deadliest, which makes it a major public health problem with 1.5 million deaths around the world (World Health Organization, 2002). The hepatic fibrogenesis appears in two phases during which the quiescent cells in the liver, or stellate cells, whose physiological role is to store vitamin A, play a crucial role. The first phase is characterized by an inflammatory phase in which the molecules from, for example, the metabolism of alcohol or the cleavage of proteins by hepatitis B or C viruses are capable of inducing the release of pro-inflammatory molecules by several cell types [6]. During this initial phase, hepatocytes activate and recruit T-cells whereas the biliary epithelial cells activate resident macrophages called Kupffer cells of the liver. The result is a production of free radicals and other soluble factors capable of stimulating hepatic stellate cells [7] and lead to their transformation. Following the first step, a second fibrotic phase where the quiescent stellate cells turn into myofibroblasts and lead to the apoptosis of hepatocytes. This induces an accumulation of fibrotic cells such activated stellate cells and myofibroblasts from fibrocytes differentiation. These cells also induce the recruitment of immune cells responsible for chronic inflammation. The fibrogenic process is associated with MMPs/TIMPs imbalance which produce excessive components of the ECM. The liver fibrosis may change in cirrhosis, characterized by the formation of regenerative nodules and a reduction in the size of the body. In 40% of cases, it is asymptomatic but may lead to complications such as portal hypertension, the liver failure and hepatocellular carcinoma [7,8].

## THE MATRIX METALLOPROTEINASES

The MMPs form a group of structurally related extracellular zinc endopeptidases known for their ability to cleave one or several constituents of the ECM [9]. MMPs are multidomain enzymes that have a pro-domain, an active domain, a zinc-binding domain and a haemopexin domain (for details see [10]). Moreover, membrane-type MMPs (MT-MMPs) contain a membrane anchor with certain MT-MMPs also possessing a cytoplasmic domain at the C-terminus. Gelatinases (MMP-2 and MMP-9) contain a gelatin-binding domain with three fibronectin-like repeats. In particular, MMP9 also contains a serine-, threonine- and proline-rich *O*-glycosylated domain. *N*-glycosylation sites, one of which is conserved among most MMPs, are indicated with a Y symbol. Part of the pro-peptide, which contains the chelating cysteine, and part of the zinc-binding domain with three histidines are indicated in one-letter code for the amino acids at the top of the figure [10]

Zymogen forms of the MMPs (pro-MMPs) are secreted into the extracellular space from a large number of cell types, where activation of the pro-MMPs in the local microenvironment can result in discrete alterations in the tissue architecture. MMP synthesis and functions are regulated by transcriptional activation, post-transcriptional processing (release of pro-domain, cell surface shedding) and control of activity by a family of endogenous inhibitors collectively known as tissue inhibitors of metalloproteinases (TIMP). Upon stimulation, many cell types including macrophages have been identified as producers of MMPs and TIMPs in a context of inflammatory process, strongly suggesting the involvement of MMPs in numerous inflammatory diseases. MMPs are not only put forward as physiological mediators of the ‘turnover’ of the ECM but are also considered to be critical factors of the remodelling processes in pathological conditions [11]. Indeed, a marked increase in their expression is observed in and associated with a variety of inflammatory diseases.

Regarding the fibrogenic process, the activities of MMPs that can degrade matrix, might be observed to be under-expressed in fibrosis or, if present, could function to resolve the excess matrix. However, some MMPs are indeed anti-fibrotic, whereas others can have profibrotic functions. MMPs modulate a range of biological processes, especially processes related to immunity and tissue repair and/or remodelling. Although we do not yet know precisely how MMPs function during fibrosis–that is, the protein substrate or substrates that an individual MMP acts on to effect a specific process–experiments in mouse models demonstrate that MMP-dependent functions during fibrosis are not limited to effects on ECM turnover. A nice summary of which MMPs are involved in the development of inhibition of fibrogenic process has been proposed by Giannandrea and Parks [12]. Although there are many mechanisms for generating fibrosis common among these organs, notably including the stable activation of myofibroblasts from resident interstitial cells and common immune features, it is important to note that the roles for specific MMPs are not necessarily the same among different organ systems [12]

MMPs are expressed at low levels in normal adult tissues, and their involvement appears to play an important role in the development of a number of pathological processes including fibrosis. Further studies have demonstrated the involvement of MMPs in the pathogenesis of pulmonary and/or hepatic fibrosis. For example, bronchoalveolar lavage fluids from individuals with sarcoidosis [13] or pulmonary fibrosis [14] contain high levels of collagenase, thought to be neutrophil-derived, a fact that has been suggested to be related to the development of fibrosis in these subjects. Fukuda et al. [15] investigated MMPs activities in the lungs of patients with bronchiolitis obliterans organizing pneumonia and IPF. In bronchiolitis obliterans organizing pneumonia, predominant MMPs, mainly active MMP-2, may constitute the mechanisms of reversibility of fibrotic changes in this disease. Conversely, a TIMP-2 increase in IPF may contribute to the stable ECM deposition and the irreversible pulmonary structural remodelling. Hayashi et al. [16] have suggested the involvement of MMP-2 in collagen deposition in IPF. Moreover, Pardo et al. [17] reported that cultured fibroblasts derived from patients with IPF exhibited an increased MMPs/collagen ratio. The involvement of MMP-2 in ECM deposition was also suggested in a model of bleomycin-induced pulmonary fibrosis in rabbits [18]. It has been recently suggested that in IPF there is a higher expression of TIMPs compared with collagenases, supporting the hypothesis that no degrading fibrillar collagen microenvironment is prevailing [1]. Such observations have been confirmed *in vivo* in a murine model of bleomycin-induced pulmonary fibrosis [19].

ECM changes in the liver depend upon ECM synthesis and MMP-mediated ECM proteolytic degradation. Healthy adult livers have a moderate ECM turnover, which seems to correlate with the relatively small amounts of MMPs constitutively detected in those livers [20]. Hepatic injury is frequently categorized into acute and chronic liver injury and MMPs have been linked to a number of acute and chronic liver disorders [21].

Chronic inflammatory process in the liver is responsible of an excessive accumulation of ECM components including collagens, and proteoglycans, which are major players in the formation of transformed tissue. MMPs and TIMPs are also the main regulators of ECM turnover in hepatic fibrosis [22]. Hepatic stellate cells, which express ECM components, MMPs and TIMPs in different timeframes are thought to play central roles in the development of hepatic fibrosis [20]. MMP-1, MMP-8 and MMP-13 seem to be among the candidates for an anti-fibrotic role, since their overexpression has been associated to significantly reduced liver fibrosis and enhanced hepatocyte proliferation [23–25]. MMP-13 involvement in the liver has been correlated with the change from normal to abnormal matrix turnover in the CCI4 preclinical injury model [26].

**Figure 1:**
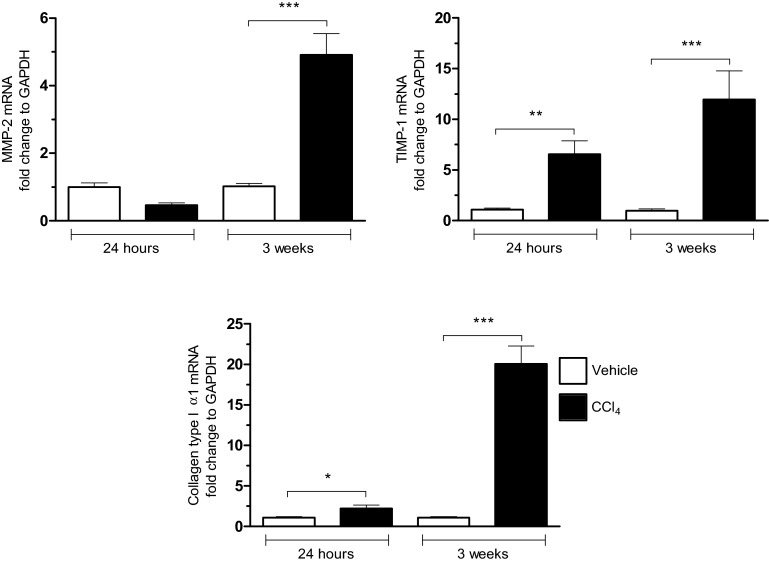
Increased expression for MMP-2, TIMP-1 and collagen 1α during the fibrogenic process in liver mRNA expressions for MMP-2, TIMP-1 and α1-collagen were measured 24 h after treatment with the CCl_4_ (1 IP injection; 0.35 mL/kg) or after 3 weeks of treatment to the CCl_4_ (6 IP injections; 0.35 mL/kg) in C57Bl/6J mice compared with controls (vehicle), (mean±S.E.M.; *P<0.05; **P<0.01; ***P<0.001).

**Figure 2:**
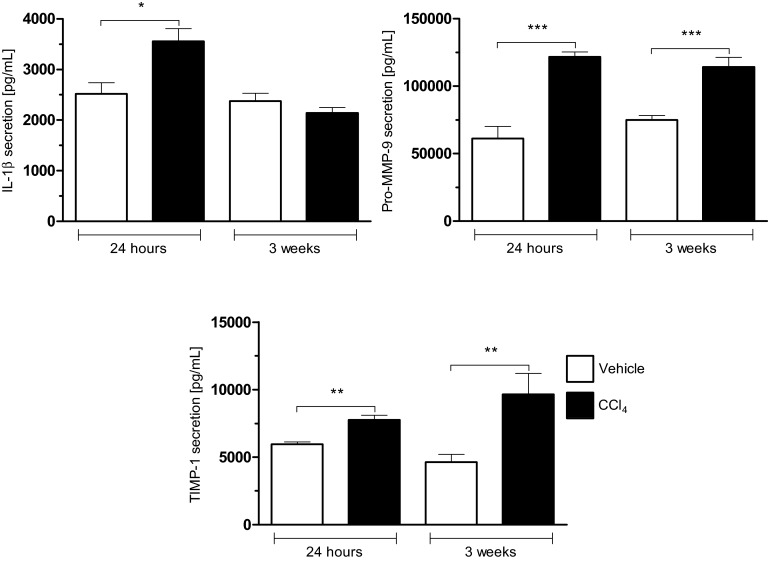
Increased production of IL-1β, Pro-MMP-9 and TIMP-1 during the fibrogenic process in liver Production of IL-1β, Pro-MMP-9 and TIMP-1 were measured 24 h after treatment with the CCl_4_ (1 IP injection; 0.35 mL/kg) or after 3 weeks of treatment to the CCl_4_ (6 IP injections; 0.35 mL/kg) in C57Bl/6J mice compared with controls (vehicle), (mean±S.E.M.; *P<0.05; **P<0.01; ***P<0.001).

Using the classical model of carbon tetrachloride (CCl_4_)-induced liver fibrosis in mice, we observed a significant increase for type I collagen α1 at 24 h and 3 weeks associated with an increase in mRNA expression for MMP2 and a release of pro MMP-9 (Figures 1 and 2). Moreover, MMP-13 gene deletion results in a retarded resolution of CCI4-induced fibrosis [27]. MMP-9 expression has been detected in the early stages of hepatic fibrogenesis and it may release/activate TGF-β, a major pro-fibrotic cytokine, from ECM reservoirs [28–30]. Additionally, MMP-9 may promote hepatic stellate cell apoptosis in the presence of low levels of TIMP-1 [29].

## EFFECT OF INHIBITION OF MMPS IN INFLAMMATORY PROCESS AND FIBROSIS

TIMPs are specific endogenous inhibitors that bind to the active site of the MMPs in a stoichiometric 1:1 molar ratio, thereby blocking access to ECM substrates. Four TIMPs (TIMP-1, -2, -3 and -4) have been identified in vertebrates, and their expression is regulated during development, tissue remodelling but also inflammation [31]. Given that MMPs degrade various components of the ECM, a tight regulation of MMP activities is essential to prevent excessive matrix degradation. The primary action of TIMPs is to inhibit MMPs, but numerous studies have reported cell growth-promoting, anti-apoptotic, steroidogenic and anti-angiogenic activities (reviewed in [32,33]), which are in part independent of MMP inhibition. Since the main cellular sources of TIMP-1 are macrophages and fibroblasts, one can easily suggest that TIMP-1 is involved in tissue remodelling associated with the activation of macrophages in inflammatory process. Studies have demonstrated that monocytes secrete large quantities of basal levels of TIMP-1, but are unresponsive to LPS, whereas macrophages secrete lower basal levels of TIMP-1, and are up-regulated by LPS [33]. It has also been speculated that TIMP-1 may be involved in the modulation of inflammatory responses and may also function to stabilize matrix components deposited in the injured lung.

Indeed, we previously reported that TIMP-1 was markedly increased in mice's lungs, 24 h after the administration of bleomycin at day 1 [34]. During this period, we were not able to observe collagen deposition, but bleomycin induced an important inflammatory reaction characterized by an influx of neutrophils and probably an increase in macrophage activity. However, the depletion of mice in neutrophils did not modify the level of the TIMP-1 protein in comparison with control mice [34]. We also reported that the non-selective MMP inhibitor, batimastat reduced the development of bleomycin-induced fibrosis in mice associated with a decrease in TIMP-1 levels in bronchoalveolar lavage fluids [35]. This strongly suggests that TIMP-1 may be considered as an available target for tissue remodelling and fibrosis.

Through the importance of ECM remodelling, there is a significant interest in using MMPs inhibition as a therapeutic strategy. However, the TIMPs have not proved to be suitable for pharmacological applications due to their short half-life *in vivo* [36]. Numerous MMP inhibitors are still under development, in spite of extensive efforts by almost all major pharmaceutical companies, indicating that the development of MMP inhibitors is very challenging [37]. The first synthetic broad-spectrum MMP inhibitor includes hydroxamic acid derived inhibitors such as BB-94 (Batimastat), BB-1101, BB-2293, BB-2516 (marimastat) and CT1746. Batimastat and marimastat, are competitive MMP-inhibitors and Zn^2+^ chelating mimickers of collagen. The hydroxamate acts as a bidentate ligand with the active-site zinc ion to form a slightly distorted trigonalbipyramidal coordination geometry. In MMP1 inhibition, the hydroxamate oxyanion forms a strong, short hydrogen bond to the carboxylate oxygen of the catalytical Glu^219^ that is orientated towards the unprimed binding regions [12].

Initial results have been promising in cancer research in blocking the progression of tumour growth [38,39]. We have previously showed that batimastat significantly limits the development of bleomycin-induced pulmonary fibrosis in mice associated with a reduction of levels of TIMP-1 [35].

Similarly to that reported for pulmonary fibrosis, there is growing evidence supporting a TIMP to degrade ECM in hepatic fibrosis [40]. TIMP-1 and TIMP-2 are expressed in high levels in murine fibrotic livers after CCI_4_ administration [41]. We also clearly observed an increase in mRNA expression and production of TIMP-1. Moreover, treatment of fibrotic murine livers with modified synthetic siRNA targeting TIMP-2 reduces fibrosis by decreasing HSC activation and collagen accumulation [42]. TIMP-1 over-expression may exacerbate liver fibrosis, and hepatic fibrosis may mediate fibrosis through the TIMP-1 dependent signalling pathway [43]. The activated HSCs not only secret excess type I collagen, but markedly increase TIMP expression, leading to a shift towards excess ECM synthesis and fibrogenesis [44]. In support of the role of TIMP-1 in vivo, transgenic mice over-expressing human TIMP-1 showed deteriorated fibrosis in response to long-term CCl_4_ administration [45]. TIMP-1 is also a contributory factor in the development of hepatic fibrosis as shown in animal models and patients [46]. Due to the inhibition of collagenase and others enzymes involved in the turn TIMP-1 alone may not induce liver fibrosis, but it can significantly exacerbate the hepatic fibrosis [47]. Another study reports that transplantation of stem cells expressing TIMP-1-shRNA is able to inhibit the progression of liver fibrosis and possibly restore the liver function in a rat model [48].

These observations support the view that therapies aimed at overexpressing selective MMPs and reducing excessive TIMP levels may ameliorate fibrosis.

## ROLE OF INFLAMMASOME PATHWAY IN THE DEVELOPMENT OF FIBROSIS

Several inflammatory diseases have been reported to be linked with the activation of inflammasome pathway including gout [49], Crohn's disease [50], rheumatoid arthritis [51] and cryopyrin-associated periodic syndrome (CAPS) [52].

The best-characterized inflammasome consists of three main components, the Nod-like receptor (NLR)-family protein, NLRP3, pro-caspase-1 and the ASC (apoptosis speck-like protein containing a CARD) adapter, which bridge interactions between the former proteins [53]. NLRP3 activation requires two signals. Cell priming with an NF-κB activator, such as the TLR4-ligand LPS, is the first step of NLRP3 inflammasome activation leading to its own involvement [54]. The second signal includes a broad variety of activators, in which one the major pathway includes the P2X_7_ purinergic receptor. Extracellular ATP, release upon cell death, is sensed by this receptor to induce a potassium efflux and the recruitment of pannexin-1, a membrane pore that allows the delivery of extracellular pathogen-associated molecule patterns (PAMPs) and danger-associated molecule patterns (DAMPs) into the cytosol. NLRP3 is expressed by myeloid cells and is then up-regulated and activated in response to the stimulation of macrophages with these PAMPs or DAMPs [55]. NLRP3 interacts with ASC and pro-caspase-1 to become effective. Following autoactivation via inflammasome assembly, caspase-1 cleaves pro-IL-1β and pro-IL-18, whose biologically active form IL-1β and IL-18 are then secreted.

IL-1β is a cytokine with major roles in inflammation, innate immune response and fibrosis. This cytokine is produced by activated monocytes, macrophages and dendritic cells, inducing the production of chemokines or cytokines such as TNF-α and IL-6, or proteases such as MMPs associated with neutrophil recruitment and proliferation of resident cells mainly fibroblasts [56]. Since mature IL-1β is very potent, its production is tightly regulated on expression, transcription and secretion [57].

It has also been previously demonstrated that pulmonary fibrosis is closely associated with the activation of NLRP3-inflammasome pathway, production of IL-1β and TIMP-1 [58,59]. Indeed, several studies using KO mice for several components of inflammasome pathway including NLRP3, ASC and caspase-1 showed a reduction of experimental pulmonary fibrosis induced by bleomycin in mice [58–60]. In an alveolar basal epithelial cell line, IL-1β was shown to stimulate transcription of TGF-β via an NF-κB and AP-1 pathway [61]. The inhibition of the collagen deposition and fibrosis is well correlated with the imbalance between MMPs and TIMP-1 in favour to increase TIMP-1 production. Moreover, production of TIMP-1 and collagen deposition is reduced in interleukin-1 (IL-1) receptor and MyD88 deficient mice [56]. Similarly, cigarette smoke-induced inflammation and elastase-induced emphysema in mice depends on inflammasome pathway and IL-1R1/MyD88 signalling [62,63]. Due to the fact that the IL-1R signals via a MyD88 domain, in common with most Toll receptors, and results in the involvement of pro-IL-1β, the release of IL-1β can initiate a positive feedback cycle with IL-1β contributing to its own production.

Abnormal cell activation may provide signal that alert the immune system to danger, triggering innate immunity activation leading to inflammatory process and remodelling. In this context, dying cells release danger signals that may activate the immune system and stimulate innate and adaptive immunity. The danger signals are recognized by membrane receptors such as TLRs [64] or cytosolic receptors such NLRP3-inflammasome [53]. It was clearly demonstrated that uric acid is a danger signal activating NLRP3-inflammasome pathway in gout arthritis [49]. Uric acid is a product of purine catabolism which is produced from injured tissue *in vivo* after tumour chemotherapy leading to tumour lysis syndrome characterized by hyperuricemia [65]. At high local concentration, uric acid precipitates and forms crystals that cause inflammation as observed in gout and activate the caspase-1, leading to the production of IL-1β. It has been demonstrated that uric acid locally produced in the lung upon bleomycin-induced DNA damage and degradation induced the activation of NLRP3-inflammasome pathway.

ATP has been described also as a danger signal activating NLRP3-inflammasome leading to the pro-inflammatory cytokine IL-1β release in lung. Extracellular ATP was shown to play a major role to trigger synthesis and release of mature IL-1β after initial stimulation of macrophages by an inflammatory signal such as LPS [59]. ATP is described as an agonist of purinergic P2 receptor predominantly expressed on immune cells [66] and is reported to be involved in the pathophysiology of LPS-induced lung injury, modulating airway inflammatory process and functional changes [67]. ATP mainly activates the P2X_7_ purinergic receptor, leading to trigger ASC-caspase-1 complex in a NLRP3-dependent manner, leading to the production of IL-1β [68]. Fibrotic patients have elevated ATP content in bronchoalveolar lavage fluid in comparison with control individuals [59]. It has been shown an early increase in ATP levels in bronchoalveolar lavage fluid on bleomycin administration in mice. Modulation of ATP levels with the ATP-degrading enzyme apyrase greatly reduced bleomycin-induced inflammatory cell recruitment, lung IL-1β and TIMP-1 production. P2X[7] receptor-deficient mice presented dramatically reduced lung inflammation, with reduced fibrosis markers such as lung collagen content, TIMP-1 and MMP-9 [59]. This clearly proposed that ATP released from bleomycin-injured lung cells constitutes a major endogenous danger signal that engages the P2X[7] receptor/pannexin-1 axis, leading to IL-1β maturation and lung fibrosis. We recently showed that ATPγS and BzATP, two analogues of ATP are able to potentiate the release of IL-1β from human monocyte-derived macrophages induced by low concentration of LPS [69]. Our recent results demonstrate the involvement of purinergic receptors and the NLRP3-inflammasome pathway in the secretion of IL-1β by MSU-stimulated human macrophages [70]. Our findings suggest that blockade of the NLRP3-inflammasome pathway or the purinergic receptors is a novel potential therapeutic approach to control the inflammatory process in several associated pathologies such as fibrosis.

**Figure 3:**
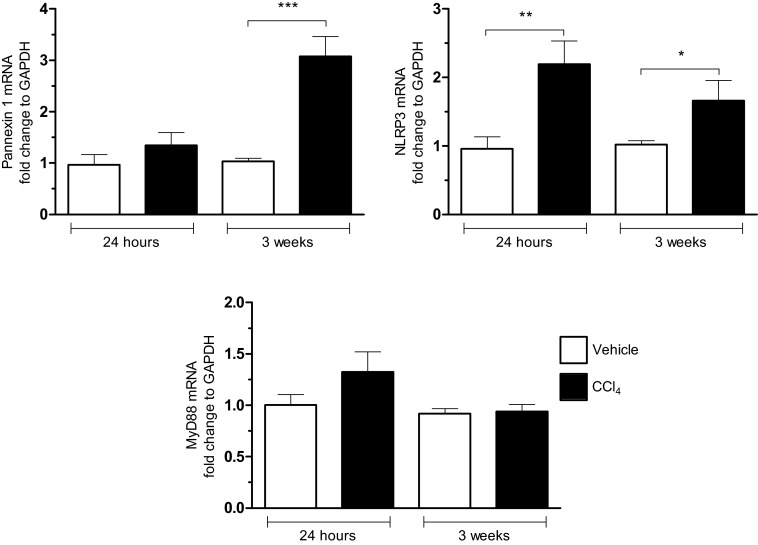
Increased expression for pannexin-1, NLRP3 and MyD88 during the fibrogenic process in liver mRNA expressions for pannexin-1, NLRP3 and MyD88 were measured 24 h after treatment with CCl_4_ (1 IP injection; 0.35 mL/kg) or after 3 weeks of treatment to the CCl_4_ (6 IP injections; 0.35 mL/kg) in C57Bl/6J mice in comparison with controls (vehicle), (mean±S.E.M.; *P<0.05; **P<0.01; ***P<0.001).

IL-1β and inflammasome pathway have been reported to play an important role in chronic liver inflammation leading to fibrosis and cirrhosis [71]. Our results showed a significant increase in NLRP3 but not of MyD88 at 24 h and 3 weeks and in pannexin-1 at 3 weeks in CCl_4_-induced liver fibrosis in mice (Figure 3). In rats, IL-1Ra administration attenuated dimethylnitrosamin (DMN)-induced liver cirrhosis [72] and during experimental liver fibrosis induced by thioacetamide (TAA) administration, IL-1 levels are elevated, where they peak on day 1 followed by a peak in type I collagen on day 3. They also demonstrate the increase in α-smooth muscle actin, indicating the myofibroblast differentiation, and there is significantly less liver fibrosis in IL-1R deficient mice [73]. Moreover, the expression of MMP-9, MMP-13 and TIMPs, are dependent upon IL-1 activation [73]; however, the exact mechanisms by which IL-1R signalling promotes fibrosis and the cell type(s) that produce(s) IL-1β remain elusive. Hepatic stellate cells expressed components of the inflammasome and activation of primary mouse stellate cells or LX-2 HSC cells with MSU resulted in increased TGF-β and collagen-1 expression, actin reorganization and inhibition of HSC chemotaxis in an NLRP3-dependent manner [74]. These changes did not occur if the HSC were lacking the inflammasome component ASC. In a CCl_4_ and TAA-induced in vivo liver fibrosis model, expression of TGF-β and collagen-1 was significantly reduced in mice lacking either NLRP3 or the adaptor molecule ASC. Using NLRP3 KO mice, it was also reported that NLRP3 inflammasome activation is required for fibrosis development in non-alcoholic fatty liver disease (NAFLD) [75].

Additional data from the CCl_4_-induced fibrosis model indicated that IL-1 receptor antagonist (IL-1Ra) protected mice from acute hepatocyte damage and promoted hepatocyte proliferation [76]. IL-1Ra was also reported to improve inflammasome-dependent alcoholic steatohepatitis in mice [77]. In an animal model of NASH, inhibition of hepatic cell death with a pan-caspase inhibitor suppressed fibrosis [78]. The specific role of caspase-1 compared with other caspases is yet to be fully understood in liver fibrosis.

**Figure 4:**
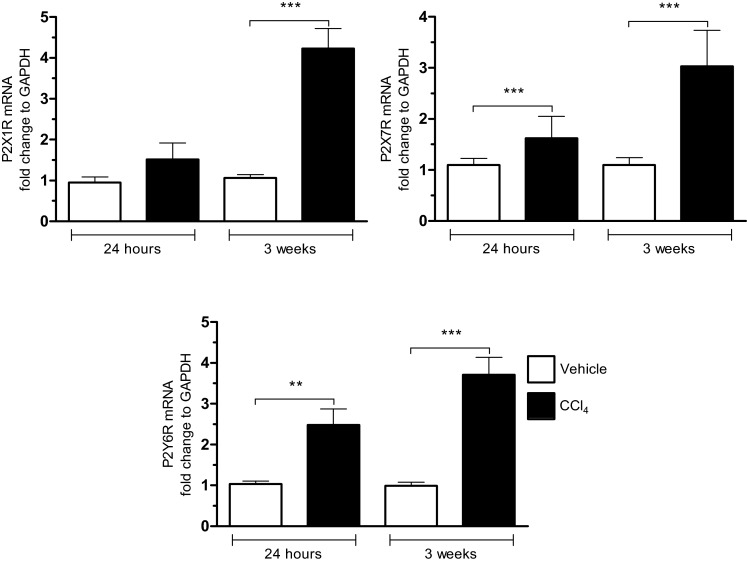
Increased expression for purinergic receptors during the fibrogenic process in liver mRNA expressions for purinergic receptors P2X1, P2X7 and P2Y6 were measured 24 h after treatment with the CCl_4_ (1 IP injection; 0.35 mL/kg) or after 3 weeks of treatment to the CCl_4_ (6 IP injections; 0.35 mL/kg) in mice C57Bl/6J in comparison with controls (vehicle), by q-PCR technique (mean±S.E.M.; *P<0.05; **P<0.01; ***P<0.001).

Purinergic signalling is also involved in the development of liver fibrosis [79]. Using the CCl_4_-preclinical experimental model of experimental liver fibrosis, we observed a significant increase in mRNA for P2X_1_ receptor at 3 weeks and in P2X_7_ and P2Y_6_ receptors at 24 h and 3 weeks (Figure 4). The involvement of P2X_7_ is supported by the study showing that P2X_7_ receptor blockade attenuates mouse liver fibrosis [80]. This is only in accordance with the fact that P2X_7_ receptor is involved in non-alcoholic steatohepatitis [81].

## CONCLUSIONS AND FUTURE DIRECTIONS

It has not yet been clearly established which MMP activity needs to be inhibited in order to have an impact on fibrosis associated with inflammatory process. Since numerous reported evidences suggest that different MMPs play an important role in the pathogenesis of tissue remodelling associated with inflammatory processes in several diseases, broad spectrum MMPs inhibitors may have therapeutic potential.

One alternative is a gene transfer to overexpress TIMPs which can reduce MMPs activity and modulate tissue remodelling. Several preclinical studies of various diseases have reported encouraging data. However, expressing wild-type TIMPs could have drawbacks because multiple MMPs may be inhibited. The best route to success is probably the development of engineered TIMPs with altered specificity, to enable the targeting of specific MMPs. One alternative of selective MMP inhibitors could be the RNA interference therapy development.

The recent characterization of the involvement of the NLRP3-inflammasome pathway has opened a large possibility of new therapeutic targets for the reduction of collagen deposition and fibrosis. However, we need of selective and available tools to validate the right target. For instance, our goal is to investigate the role of purinergic receptors. Regarding the recent data, P2X_7_ receptor would be a good candidate. However, it is not excluded that blockade of one type of receptors may induce a compensation by others. The screening of potential drugs effective in preclinical models of fibrosis would be the next challenge.

## Abbreviations

ASC: apoptosis speck-like protein containing a CARD
CCl_4_: carbon tetrachloride
DAMP: danger-associated molecule pattern
ECM: extracellular matrix
IL-1: interleukin-1
IL-1Ra: IL-1 receptor antagonist
IPF: Idiopathic pulmonary fibrosis
MMP: metalloproteinase
MT-MMP: membrane-type MMP
NLR: Nod-like receptor
PAMP: pathogen-associated molecule pattern
TAA: thioacetamide
TIMP: tissue inhibitors of metalloproteinase
